# Biomarkers for the diagnosis and management of heart failure

**DOI:** 10.1007/s10741-021-10105-w

**Published:** 2021-04-14

**Authors:** Vincenzo Castiglione, Alberto Aimo, Giuseppe Vergaro, Luigi Saccaro, Claudio Passino, Michele Emdin

**Affiliations:** 1 Institute of Life Sciences, Scuola Superiore Sant’Anna, Pisa, Italy; 2 Fondazione Toscana Gabriele Monasterio, Pisa, Italy

**Keywords:** Biomarkers, Heart failure, Natriuretic peptides, Troponin, SST2, Inflammation

## Abstract

Heart failure (HF) is a significant cause of morbidity and mortality worldwide. Circulating biomarkers reflecting pathophysiological pathways involved in HF development and progression may assist clinicians in early diagnosis and management of HF patients. Natriuretic peptides (NPs) are cardioprotective hormones released by cardiomyocytes in response to pressure or volume overload. The roles of B-type NP (BNP) and N-terminal pro-B-type NP (NT-proBNP) for diagnosis and risk stratification in HF have been extensively demonstrated, and these biomarkers are emerging tools for population screening and as guides to the start of treatment in subclinical HF. On the contrary, conflicting evidence exists on the role of NPs as a guide to HF therapy. Among the other biomarkers, high-sensitivity troponins and soluble suppression of tumorigenesis-2 are the most promising biomarkers for risk stratification, with independent value to NPs. Other biomarkers evaluated as predictors of adverse outcome are galectin-3, growth differentiation factor 15, mid-regional pro-adrenomedullin, and makers of renal dysfunction. Multi-marker scores and genomic, transcriptomic, proteomic, and metabolomic analyses could further refine HF management.

Heart failure (HF) is a syndrome characterized by the inability of the heart to pump enough blood and oxygen to support the metabolic demands of other organs [1]. HF affects around 64 million of patients worldwide, and its prevalence is increasing due to population ageing, the growing burden of comorbidities and risk factors for HF, and the longer survival after myocardial infarction [2].

HF is commonly classified based on left ventricular ejection fraction (LVEF) into the categories of HF with preserved (HFpEF; LVEF ≥ 50%), mid-range (HFmrEF; LVEF 40–49%), and reduced ejection fraction (HFrEF; LVEF < 40%) [2]. HFpEF is predominantly characterized by diastolic dysfunction and often results from heart damage due to comorbidities (e.g., obesity, chronic kidney disease (CKD), chronic obstructive pulmonary disease) or accumulation disorders (e.g., cardiac amyloidosis). HFrEF, on the other hand, is characterized prevalently by systolic dysfunction, secondary to a direct heart damage (such as an acute coronary syndrome), a cardiomyopathy or a valve disease [2]. While the pathophysiology of HFpEF is likely multifactorial, an imbalance in the neuroendocrine systems regulating cardiovascular homeostasis plays a central role in HFrEF. The sympathetic nervous system (SNS) and the renin–angiotensin–aldosterone system (RAAS) are overactivated and are not adequately counterbalanced by the increased release of natriuretic peptides (NPs). This imbalance promotes structural alterations leading to progressive hypertrophy and dilation of cardiac chambers. These alterations have detrimental effects on cardiac pump function, and ultimately lead to symptom development, as well as an increased susceptibility to arrhythmias and cardiac conduction disorders [1].

Thanks to the introduction of pharmacological therapies (beta-blockers, angiotensin-converting enzyme (ACE) inhibitors or angiotensin receptor blockers, mineralocorticoid receptor antagonists, neprilysin inhibitors, and, recently, sodium-glucose 2 co-transport inhibitors) and devices (implantable cardioverter defibrillator and cardiac resynchronisation therapy), the survival of HF patients has progressively improved. A careful use of biomarkers might help refine the management of patients with HF and further improve their prognosis.

## Characteristics of an ideal biomarker

The term “biomarker” (from “biological marker”) was coined in 1989 to identify a “measurable and quantifiable biological parameter used to assess the health and physiology of patients in terms of disease risk and diagnosis” [3]. In 2001, the National Institute of Health defined a biomarker as “a characteristic that is objectively measured and evaluated as an indicator of normal biological processes, pathogenic processes, or pharmacologic responses to a therapeutic intervention” [4]. A further definition by the World Health Organization states that “a biomarker is any substance, structure or process that can be measured in the body or its products and influence or predict the incidence of outcome or disease” [5].

Morrow and de Lemos summarized the characteristics of clinically useful biomarkers [6]. Ibrahim and Januzzi then adapted these criteria to the specific setting of HF: (1) the biomarker should be measured accurately; (2) the assay should be easily available and be interpretable at a reasonable cost, results should be available quickly, biological variation should be defined, imprecision should be low, reference limits should be well defined, possible pre-analytical, analytical and post-analytical sources of error well known; (3) a new biomarker should explore an important disease pathway in HF; (4) the analyte of interest should provide information that is not available through objective examination and laboratory investigation; (5) a new biomarker should guide the diagnosis, risk stratification, or management of HF [7].

Biomarkers can serve as “antecedent biomarkers” (predicting future disease development), screening biomarkers (identifying subclinical disease), diagnostic biomarkers (recognizing clinically manifest disease), staging biomarkers (defining disease severity), prognostic and therapeutic biomarkers (predicting disease evolution and the response to therapy, respectively), as well as inclusion/exclusion and outcome criteria for clinical trials [1, 3] (Table 1).

**Table 1:** Characteristics of an “ideal” biomarker and possible types of biomarkers in heart failure

Characteristics of an “ideal” biomarker	Types of biomarkers
• Has been thoroughly tested • Is cheap, easily measured and interpreted, with well-known characteristics • Reflects a pivotal pathophysiological pathway • Provides additional information to those already available • Allows a better definition of heart failure diagnosis, prognosis or management	• Antecedent biomarker • Screening biomarker • Diagnostic biomarker • Staging biomarker • Prognostic biomarker • Treatment response biomarker • Surrogate endpoint

Although imaging findings, signals, functional tests and genetic variants can all be defined as “biomarkers,” this review will focus on circulating biomarkers [1]. Since Braunwald’s first studies in the 1950s on C-reactive protein (CRP) in HF [8], hundreds of molecules have been studied, but only B-type natriuretic peptide (BNP) and N-terminal pro–B-type natriuretic peptide (NT-proBNP) come close to the characteristics of “ideal” HF biomarkers, and are often regarded as the reference standard against which other potential biomarkers must be evaluated.

## Natriuretic peptides

The discovery of atrial natriuretic peptide (ANP) in 1981 [9] and then BNP [10] showed that the heart has an endocrine function, and paved the way to the characterization of these molecules as HF biomarkers.

### BNP, NT-proBNP

**Fig. 1:**
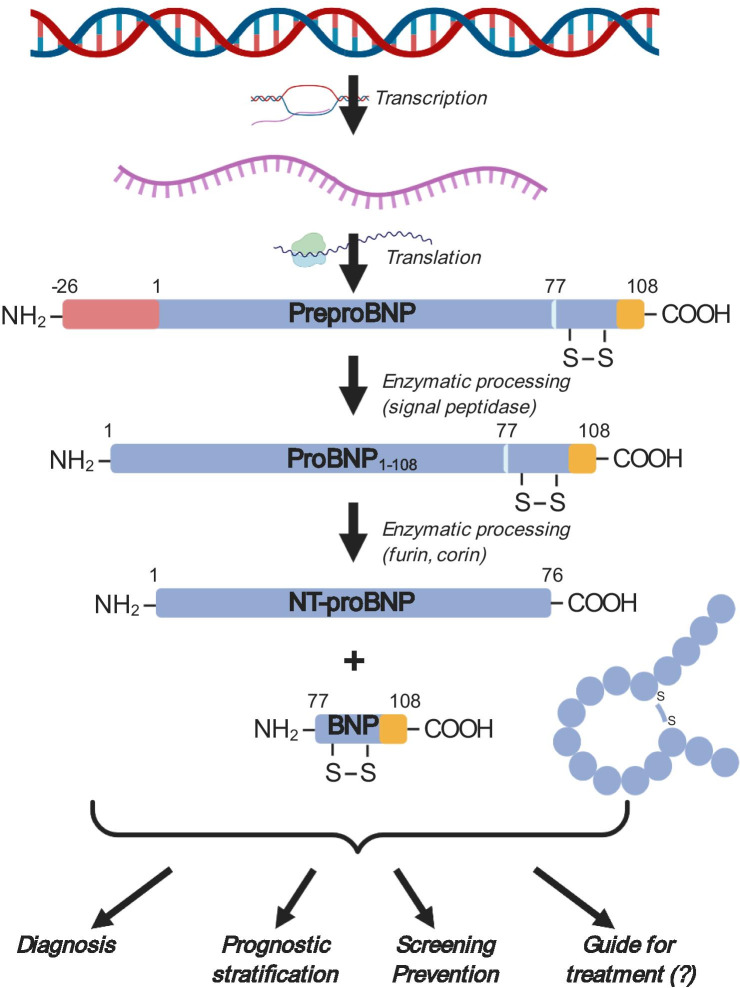
Processing of type B natriuretic peptides and their role as biomarkers in heart failure. BNP B-type natriuretic peptide, NT-proBNP N-terminal pro-B-type natriuretic peptide

BNP and NT-proBNP are synthesized from a pre-hormone of 134 amino acids, encoded by the *NPPB* gene. Once a residue of 26 amino acids is cleaved, BNP_1-108_ is produced, which is converted by the enzymes furin or corin into BNP_1-32_, the biologically active molecule, and NT-proBNP_1-76_, its inactive N-terminal fragment (Fig. 1). BNP is produced primarily by ventricular cardiomyocytes in response to volume or pressure overload [11]. Circulating BNP and NT-proBNP levels are normally very low, but increase significantly in HF patients as a mechanism to restore normal hemodynamics. BNP promotes arterial vasodilation, dieresis, and natriuresis, exerts anti-hypertrophic and anti-fibrotic effects, and counteracts the activation of RAAS, SNS and the endothelin systems [11]. BNP binds to NP receptor A (NPR-A) and NPR-B, which have guanylate-cyclase activity. Around 25% of BNP is excreted unmodified by the kidneys. The remaining part is eliminated after binding to the NPR-C receptor or through enzymatic degradation by neprilysin. Conversely, NT-proBNP has only a passive excretion, mainly by the kidney. Due to their different clearance, NT-proBNP has a longer half-life (120 vs. 20 min) and higher plasma concentrations (approximately 6 times) than BNP [11].

#### Diagnosis

In the Breathing Not Properly study, which included 1586 patients admitted to the emergency room for new-onset dyspnoea, BNP levels were significantly higher in subjects with acute HF, and plasma BNP increased in parallel with the New York Heart Association (NYHA) class. BNP had an area under the curve (AUC) of 0.91 for the diagnosis of HF; a cutoff of 100 ng/L showed a better diagnostic performance (90% sensitivity, 76% specificity, 83% accuracy) than previous diagnostic scores (National Health and Nutrition Examination Survey and Framingham criteria) [12]. Similar results were reported for NT-proBNP in the N-terminal Pro-BNP Investigation of Dyspnea in the Emergency department (PRIDE) study, evaluating 600 patients admitted to the emergency department for dyspnoea. NT-proBNP < 300 ng/L ruled out effectively acute HF (99% negative predictive value [NPV]), and rule-in cutoffs of 450 ng/L in subjects with < 50 years (93% sensitivity, 95% specificity, 95% accuracy) and 900 ng/L in subjects with ≥ 50 years (91% sensitivity, 80% specificity, 85% accuracy) were proposed [13]. Indeed, NP levels tend to increase in the elderly, probably because of age-dependent decreases in left ventricular (LV) compliance and kidney function. The use of age-specific cutoffs was supported by the International Collaborative study of NT-proBNP (ICON), where the use of different thresholds by age groups (≥ 450, ≥ 900, and ≥ 1800 ng/L in subjects aged < 50, 50–75, and > 75 years) increased the diagnostic performance of NT-proBNP [14].

HF outpatients have usually lower NP levels. In this setting, BNP and NT-proBNP were assessed mostly to rule out HF, with cutoffs that maximized NPV [15]. Age-stratified thresholds of NT-proBNP have been proposed also in chronic HF [16].

American College of Cardiology/American Heart Association (ACC/AHA) guidelines recommend the use of BNP and NT-proBNP to diagnose HF (class I, level of evidence (LOE) A), without indicating specific threshold values [17]. Conversely, European Society of Cardiology (ESC) guidelines recommend the use of BNP and NT-proBNP for the exclusion of HF (class IIa, LOE C), with reference values < 100 ng/L and < 300 ng/L for acute HF, respectively, and < 35 ng/L and < 125 ng/L for chronic HF, respectively [2] (Table 2). Higher NP levels support the diagnosis of HF, but further investigation is required to confirm the diagnosis. A recent ESC position paper on the use of NPs proposes both rule-out and rule-in cutoffs for BNP and NT-proBNP, the latter with age-stratified values in acute HF [18] (Table 3).

**Table 2:** Indications for the use of biomarkers in the ACC/AHA 2017 and ESC 2016 guidelines for heart failure management

Biomarker	Guidelines	Indication	Recommendation class	Level of evidence
BNP or NT-proBNP	ESC	Diagnosis	I	A
	ACC/AHA		IIa	C
	ACC/AHA	Prognostic stratification at admission (acute HF)	I	A
		Prognostic stratification at discharge (acute HF)	IIa	B
		Screening/prevention	IIa	B
MR-proANP	ESC	Diagnosis	I	A
hs-TnI/T	ESC	Diagnostic support (acute HF)	I	C
	ACC/AHA	Prognostic stratification at admission (acute and chronic HF)	I	A
sST2, galectin-3	ACC/AHA	Prognostic stratification (acute and chronic HF)	IIb	B

*ACC/AHA* American College of Cardiology/American Heart Association, *BNP* brain natriuretic peptide, *ESC* European Society of Cardiology, *hs-TnI/T* high-sensitivity-troponin I/T, *MR-proANP* mid-regional pro-atrial natriuretic peptide, *NT-proBNP* N-terminal pro-B-type natriuretic peptide, *sST2* soluble suppression of tumorigenesis-2

**Table 3:** Natriuretic peptides cutoffs for acute heart failure diagnosis

	BNP (ng/L)	NT-proBNP (ng/L)			MR-proANP (ng/L)
Chronic heart failure					
Heart failure unlikely	< 35		< 125		
“Grey area”	35–150		125–600		
Heart failure likely	> 150		> 600		
Acute heart failure		Age < 50	Age 50–75	Age > 75	
Heart failure unlikely	< 100	< 300	< 300	< 300	< 120
“Grey area”	100–400	300–450	300–900	300–1800	
Heart failure likely	> 400	> 450	> 900	> 1800	

*BNP* brain natriuretic peptide, *MR-proANP* mid-regional pro-atrial natriuretic peptide, *NT-proBNP* N-terminal pro-B-type natriuretic peptide

#### Risk stratification

Numerous studies have demonstrated that NPs are useful to stratify the risk of HF patients. In the Acute Decompensated Heart Failure National Registry (ADHERE), which included 48,629 patients with acute HF, an association between BNP entry levels and intra-hospital mortality emerged. This relationship was independent of other clinical and laboratory parameters (such as age, gender, systolic blood pressure, heart rate, dyspnea at rest, sodium, creatinine, and urea levels), and the HFrEF vs. HFpEF status [19]. A systematic review of 19 studies revealed that the relative risk of all-cause death increases by 35% for each 100 ng/L increase in admission BNP (21). Similarly, NT-proBNP can predict the short- and long-term prognosis in patients with acute HF [14, 20].

Discharge NPs seem more effective than admission values for risk stratification, as demonstrated in an analysis of 7039 patients with acute HF enrolled in the Organized Program to Initiate Lifesaving Treatment in Hospitalized Patients with Heart Failure (OPTIMIZE-HF), where discharge BNP was more predictive of 1-year death (hazard ratio [HR] 1.34 [95% confidence interval, 1.28–1.40]) and 1-year death or hospitalization (HR 1.15 [1.12–1.18]) than admission BNP [21]. ACC/AHA guidelines recommend BNP or NT-proBNP measurement on admission (class I, LOE A) and before discharge (class IIa, LOE B) for risk stratification [17].

The variation in NP levels from admission to discharge is also useful for risk prediction. In a study on 241 patients, a < 50% reduction in NT-proBNP predicted a higher risk of death or hospitalization at 1 year than a ≥ 50% reduction (HR 1.57 [1.08–2.28]), regardless of admission NT-proBNP, duration of hospital stay, and admission LVEF [22]. Even changes in NP concentrations over a few days have prognostic value: for example, a ≥ 30% BNP reduction within 5 days of initiation of inotropic support predicted all-cause mortality at 1 and 3 months [23].

In chronic HF, both BNP and NT-proBNP are important outcome predictors. In a sub-analysis of the Valsartan Heart Failure Trial (Val-HeFT) study, baseline NT-proBNP was a stronger predictor of mortality or HF hospitalization than BNP [24]. A study of 2364 patients with chronic HF found that baseline NT-proBNP predicts mortality at 1 and 5 years also in individuals aged > 77 years and those aged > 85 years, with higher cutoffs than in the general population [25]. Even in chronic HF, NP changes are more effective outcome predictors than individual values, as evidenced by another sub-analysis of the Val-HeFT study, where NT-proBNP changes at 4 months were more predictive of all-cause death than baseline values [26]. Several studies also showed that the NP changes over time are associated with LV remodelling [27]. For example, a sub-analysis of the Guiding Evidence Based Therapy Using Biomarker Intensified Treatment (GUIDE-IT) study found that, after HF therapy optimization, LVEF improved and LV volumes decreased proportionally to NT-proBNP decrease [28].

#### Screening of heart failure

In an analysis of 3346 subjects without clinical evidence of HF followed for an average of 5 years, baseline BNP or NT-proBNP > 80th percentile were associated with a significantly higher risk of new-onset HF (BNP: HR 3.07 [1.51–6.26], *p* = 0.002; NT-proBNP: HR 5.02 [2.32–10.85], *p* < 0.001) [29]. Similarly, in a sub-analysis of the Prevention of Events With Angiotensin-Converting Enzyme Inhibition (PEACE) study on 3761 subjects with stable coronary artery disease and preserved LVEF, BNP, and NT-proBNP were strong predictors of HF development during a median 5-year follow-up [30]. Dynamic changes in NPs are effective in predicting HF onset. In the Cardiovascular Health Study (CHS), NT-proBNP values were dosed at baseline and 2–3 years later in 2975 elderly subjects with no evidence of HF. After a median 12-year follow-up, among subjects with baseline NT-proBNP < 190 ng/L, those who showed a biomarker increase > 25% between the two measurements had a higher risk of developing HF (HR 2.13 [1.68–2.71]) or die of cardiovascular causes (HR 1.91 [1.43–2.53]) compared to those with stable NT-proBNP values [31]. Similarly, in the group of subjects with baseline NT-proBNP ≥ 190 ng/L, those where NT-proBNP levels increased > 25% were at a higher risk of HF (HR 2.06 [1.56–2.72]) or cardiovascular death (HR 1.88 [1.37–2.57]) [31].

Two randomized controlled trials investigated the possibility of preventing HF development through a follow-up and therapy choices based on NP levels. In the study St. Vincent’s Screening to Prevent Heart Failure (STOP-HF), 1374 subjects aged > 40 years and with at least one risk factor (hypertension, dyslipidemia, obesity, vascular disease, diabetes mellitus) or cardiovascular comorbidity (moderate-to-severe valve disease, arrhythmia requiring intervention) were randomized to a follow-up by the general practitioner or to a cardiological evaluation with echocardiography whenever BNP was > 50 ng/L. After an average of 4 years, significantly fewer subjects belonging to the second group had reached the primary endpoint of LV dysfunction with or without HF symptoms (odds ratio (OR) 0.55 [0.37–0.82], *p* = 0.003). This result is partly explained by an increased prescription of RAAS inhibitors (56.5% vs. 49.6%, *p* = 0.001) in subjects with BNP-driven follow-up and, possibly, by an increased adherence to therapy and lifestyle recommendations [32]. Similarly, in the NT-proBNP Guided Primary Prevention of CV Events in Diabetic Patients (PONTIAC) study, enrolling 300 patients with type 2 diabetes and NT-proBNP > 125 ng/L with no structural heart disease, a strategy based on rapid titration of RAAS inhibitors and beta-blockers significantly reduced the primary endpoint of hospitalization or death from cardiovascular causes at 2 years (HR 0.35 [0.13–0.98], *p* = 0.04) compared to standard follow-up [33]. Based on this evidence, ACC/AHA guidelines suggest that NPs should be used to screen subjects at risk of developing HF to optimise medical therapy and prevent LV dysfunction (class IIa, LOE B). Nonetheless, proposing a standardised biomarker-driven screening and intervention strategy is difficult due to the heterogeneous definition of “individuals at risk” in the different studies [17].

#### Guide to therapy

Guideline-recommended HF therapies tend to reduce NP levels, and changes in plasma NPs may be used as surrogate end-points to assess the efficacy of new HF therapies [34] Despite these premises, the use of NPs to guide HF therapy is still under discussion. Murdoch et al. were the first to propose an optimization of HF therapy driven by NPs. In a trial of 22 patients with chronic HF randomized to ACE-inhibitor titration guided by serial BNP measurements or empirical titration, the first approach resulted in a greater reduction in heart rate and BNP values as well as an increase in plasma renin activity after 8 weeks, reflecting a more intensive RAAS inhibition [35]. Afterwards, several small randomized controlled trials tested whether a titration strategy guided by BNP or NT-proBNP was superior to the standard of care, with positive results in some trials and neutral results in others [34]. Meta-analyses on these studies showed that a NP-guided treatment is associated with lower rates of all-cause mortality and HF hospitalization [36–38]. In the GUIDE-IT trial, 1100 patients with LVEF < 40%, a previous HF episode during the last 12 months and BNP > 400 ng/L or NT-proBNP > 2000 ng/L in the previous 3 months were randomized to a titration strategy guided by NT-proBNP (target NT-proBNP < 1000 ng/L) or the standard of care. The study was discontinued for futility after the enrolment of 894 patients, with a median follow-up of 15 months, because of no difference in the primary endpoint (cardiovascular death or HF hospitalization: HR 0.98 [0.79–1.22], *p* = 0.88). None of the secondary endpoints (individual components of the primary endpoint, all-cause death, HF hospitalizations, days to hospitalization for cardiovascular causes, adverse events) differed significantly between the two groups [39]. Furthermore, NT-proBNP-driven management did not reduce healthcare costs nor improved the quality of life [40]. However, the percentage of subjects who had reached the target of NT-proBNP < 1000 ng/L and the percentage of subjects on optimal medical therapy did not differ significantly between groups, differently from other studies, suggesting a greater therapeutic effort in the control group as well [39]. These results support the notion that we should not using NPs as an “intellectual crutch to remind physicians to practice optimal medicine” [41].

### MR-proANP

Plasma ANP increases in HF in response to the stretching of atrial cardiomyocytes. The dosage of ANP is complicated by its short half-life (2–5 min) because of rapid cleavage by neprilysin. Its precursor (proANP), produced equimolarly to ANP, has a longer half-life, and there is a reliable assay that measures its mid-regional portion (MR-proANP) [11]. In the study Biomarkers in Acute Heart Failure (BACH), MR-proANP was non-inferior to BNP to diagnose acute HF in subjects with new-onset dyspnoea (cutoff ≥ 120 pmol/L; sensitivity 97%, specificity 60%, accuracy 74%) [42]. Similarly, MR-proANP showed an only slightly lower performance than NT-proBNP to diagnose acute HF in the PRIDE study (AUC 0.90 vs. 0.94, *p* = 0.001) [43]. In both studies, MR-proBNP had an additive diagnostic value over BNP or NT-proBNP [42, 43]. ESC guidelines recommend the use of MR-proANP (as an alternative to BNP or NT-proBNP) for discrimination of acute HF from non-cardiogenic causes of dyspnoea (class I, LOE A) [2].

Several studies have also demonstrated a role of MR-proBNP in predicting death risk in acute [43] and chronic [44, 45] HF, independently of NT-proBNP. A high degree of correlation between MR-proANP and NT-proBNP was reported, such as *r* = 0.80 in a study by von Healing et al. [44].

### Factors affecting NP values

NP values can be affected by age, gender, ethnicity and genetic variants, and also by numerous cardiac and non-cardiac disorders (Table 4).

**Table 4:** Main confounding factors in the clinical interpretation of natriuretic peptides

Factors that increase natriuretic peptides concentrations	Factors that decrease natriuretic peptides concentrations
Advanced age Neprilysin inhibitor therapy* Kidney disease Cardiotoxic drugs Acute coronary syndrome Right ventricular dysfunction Pulmonary hypertension Pulmonary embolism Arrhythmias (atrial fibrillation) Anemia/conditions with hyperdynamic circulation (sepsis, hyperthyroidism)	Obesity Acute (flash) pulmonary oedema Constrictive pericarditis Cardiac tamponade

^*^Only for brain natriuretic peptide (BNP)

NP levels are slightly higher in women than men in age-matched healthy individuals [46], perhaps because of an effect of sexual hormones [47]. This difference is also found in HF patients, being more evident in HFrEF than in HFpEF [48].

NT-proBNP levels tend to be lower in black individuals. In the Dallas Heart study, which included 3148 individuals (51% Black, 31% White, 18% Hispanic) without major cardiovascular diseases (myocardial infarction, HF, or stroke), median NT-proBNP levels were significantly lower (*p* < 0.0001) in Black subjects (24 [10–52] ng/L) compared to Hispanics (30 [14–59] ng/L) and Whites (32 [16–62] ng/L) [49]. Interestingly, Blacks showed also a higher prevalence of hypertension and a higher LV mass index, which might be a consequence of their relative NP “deficiency” [49]. In another multicentric study on 92,072 patients hospitalized for HF, median BNP concentration on admission was found to be higher in Asian (1066 ng/L) and Black (866 ng/L) patients than in Whites (776 ng/L) and Hispanics (737 ng/L); nonetheless, increased BNP levels were predictive of higher in-hospital mortality and hospital length of stay irrespective of ethnicity [50].

Genetic polymorphisms can also influence NP levels. Among 11,361 subjects enrolled in the Atherosclerosis Risk in Communities (ARIC) prospective cohort study, carriers of the rs198389 GG genotype (a functional variant in the promoter region of *NPPB*) showed 41% higher mean plasma NT-proBNP concentration compared with the AA genotype, with intermediate values in heterozygotes; this difference was independent of Black or White ethnicity [51]. Notably, carriers of the GG allele had also lower incidence of hypertension, and, after a median follow‐up of 23 years, showed a lower cardiovascular mortality and longer life expectancy than carriers of the AA variant [51]. Similarly, individuals with the *NPPA* polymorphisms rs5068 or rs198358 have increased circulating levels of NPs, and a 15% reduced risk of developing hypertension [52].

Subjects with a glomerular filtration rate (GFR) < 60 mL/min/1.73 m^2^ tend to have higher NPs because of a reduction in renal clearance and cardiac damage secondary to CKD. Therefore, in patients with GFR < 60 mL/min/1.73 m^2^, the use of a higher BNP cutoff (200 ng/L) has been proposed [53]. Conversely, the adoption of age-stratified NT-proBNP cutoffs is sufficient given the correlation between age and the degree of renal dysfunction [18].

Arrhythmias, particularly atrial fibrillation, may cause a paroxysmal or sustained increase in plasma NP levels due to a release of NPs by atrial cardiomyocytes [18]. Increased wall stress is the mechanism underlying NP release in hyperdynamic conditions (such as anemia, sepsis, and hyperthyroidism), as well as in acute coronary syndromes, pulmonary hypertension, and right ventricular dysfunction [18].

Neprilysin is an enzyme degrading BNP. Neprilysin inhibitors such as sacubitril (used in combination with valsartan in subjects with HFrEF) tend to transiently increase BNP levels after the start of treatment, while NT-proBNP is not affected by this mechanism and tends to decrease in response to sacubitril/valsartan therapy. Therefore, NT-proBNP is the biomarker of choice in patients receiving sacubitril/valsartan [18, 54].

NP levels are lower in obese than non-obese subjects regardless of whether HF is present or not. This might depend from a number of factors, including reduced NP release, and different kinetics of circulating NPs. The use of 50% lower BNP and NT-proBNP cutoffs has been proposed to diagnose HF [18]. These biomarkers retain a prognostic role in obese HF patients, although their prognostic performance seems lower than other biomarkers such as high-sensitivity troponin T (hs-TnT) and soluble suppression of tumorigenesis-2 (sST2) [18, 55].

Patients with cardiac tamponade, constrictive pericarditis, or acute pulmonary edema have sometimes disproportionately low NPs compared to their symptoms. This is due to the absence of a marked increase in LV wall stress in these acute conditions and/or the rapid clinical deterioration with little time for NP production and release [18].

## Biomarkers of neurohormonal activation

**Fig. 2:**
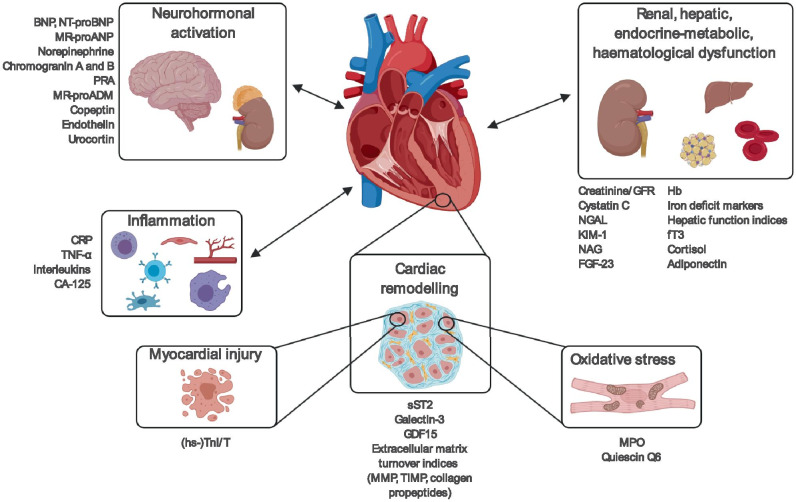
Main pathophysiological pathways involved in heart failure and their most representative biomarkers. BNP brain natriuretic peptide, CA125 cancer antigen 125, CRP C-reactive protein, FGF-23 fibroblast growth factor-23, fT3 triiodothyronine, GDF15 growth differentiation factor, GFR glomerular filtration rate, hs-TnI/T high sensitivity-troponin I/T, KIM-1 kidney injury molecule-1, MMP matrix metalloproteases, MPO myeloperoxidase, MR-proADM mid-regional pro-adrenomedullin, MR-proANP mid-regional pro-atrial natriuretic peptide, NAG N-acetyl-β-(D)-glucosaminidase, NGAL neutrophil gelatinase-associated lipocalin, NT-proBNP N-terminal pro-B-type natriuretic peptide, PRA plasma renin activity, sST2 soluble suppression of tumorigenesis-2, TIMP tissue inhibitor of metalloproteinase, TNF-α tumor necrosis factor alpha

Figure 2 and Table 5 provide an overview of HF biomarkers on the basis of the relevant pathophysiological pathway, according to the classification proposed by Braunwald in 2008 [56].

**Table 5:** Heart failure biomarkers

Neurohormonal activation	
Natriuretic peptides (*BNP*, *NT-proBNP*, ANP, *MR-proANP*) *Norepinephrine* *Chromogranin A and B* Renin/*PRA* Angiotensin II Aldosterone Adrenomedullin/*MR-proADM*	Vasopressin/*copeptin* *Endothelin* (ET-1, big ET-1, CT-proET-1) *Urocortin* Neprilysin Neuregulin CD146
Myocardial injury	
*(hs-)TnT/I* CK-MB Myosin light chain 1 hFABP	HSP-60 sFAS sTRAIL
Cardiac remodelling	
*sST2* *Galectin-3* *GDF15* *MMP* (2, 3, 4, 8, 9) *TIMP* (1, 4) *Collagen propeptides* (PIIINP, ICTP)	Myostatin Syndecan-4 Osteopontin IGFBP7 α1-antitrypsin
Inflammation	
*CRP* *TNF-α* *Interleukin* (1, 2, 6, 8, 10, 18) *CA-125* Procalcitonin LP-PLA_2_ TWEAK Fas/APO-1 Osteoprotegerin	sTNFR-1 and -2 YKL-40 IL-1RA LRG Soluble endoglin Serin protease PR3 Complex S100A8/A9 Pentraxin-3 Midkine
Oxidative stress	
*MPO* *Quiescin Q6* Oxidized LDL Urinary biopyrrins	Urinary and plasmatic isoprostanes Urinary 8-OHdG Plasmatic malondialdehyde
Comorbidity	
Renal function and injury *Creatinine/GFR* Plasma albumin, albuminuria Urinary albumin/creatinine ratio *Cystatin C* *NGAL* *KIM-1* *NAG* *FGF-23* β-trace protein β2-microglobulin Hepatic function AST, ALT GGT Bilirubin	Hematological parameters *Hemoglobin* *Iron deficiency (ferritin, transferrin saturation)* RDW Endocrine-metabolic changes *fT3*, fT4, TSH *Cortisol* *Adiponectin* Orexin Leptin Resistin IGF-1, GH
miRNA	

The biomarkers underlined are those most studied in heart failure and whose role has been thoroughly discussed in the text. ALT, alanine aminotransferase; ANP, atrial natriuretic peptide; APO-1; apoptosis 1 antigen; AST, aspartate aminotransferase; BNP, brain natriuretic peptide; CA-125, cancer antigen 125; CK-MB, creatin kinase MB; CRP, C-reactive protein; CT-proET-1, C-terminal proendothelin-1; ET-1, endothelin-1; FGF-23, fibroblast growth factor 23; fT3, triiodothyronine; fT4, thyroxine; GDF15, growth differentiation factor; GFR, glomerular filtration rate; GGT, gamma-glutamyl transferase; GH, growth hormone; hFABP, heart-type fatty acid binding protein; HSP-60, thermal shock protein 60; hs-TnI/T, high-sensitivity-troponin I/T; ICTP, collagen C-telopeptide type I; IGFBP7, insulin-like growth factor binding protein-7; IGF-1, insulin-like growth factor 1; IL-1RA, interleukin receptor antagonist 1; KIM-1, renal injury molecule 1; LP-PLA_2_, lipoprotein-associated phospholipase A2; LRG, leucine-rich alpha 2 glycoprotein; miRNA, microRNA; MMP, matrix metalloproteinase; MPO, myeloperoxidase; MR-proADM, mid-regional pro-adrenomedullin; MR-proANP, mid-regional pro-atrial natriuretic peptide; NAG, N-acetyl-β-(D)-glucosaminidase; NGAL, neutrophil gelatinase-associated lipocalin; NT-proBNP, N-terminal pro-B-type natriuretic peptide; 8-OHdG, 8-hydroxy-2′-deoxyguanosin; PIIINP, procollagen peptide type III-N-terminal; PRA, plasma renin activity; RDW, red blood cell distribution width; sFAS, soluble fragment stimulating apoptosis; sST2, soluble suppression of tumorigenesis-2; sTRAIL, soluble tumour necrosis factor-related apoptosis-inducing ligand; TIMP, tissue inhibitor of metalloproteinase; sTNFR, soluble tumour necrosis factor alpha receptor; TNF-α, tumour necrosis factor alpha; TSH, thyroid stimulating hormone; TWEAK, tumour necrosis factor-like weak inducer of apoptosis; YKL-40, chitinase-3-like protein 1

## Norepinephrine and chromogranin

In 1984, Cohn et al. reported that plasma norepinephrine was a good outcome predictor in HF [57]. This was confirmed in early HF drug trials [58], while a more recent study showed that norepinephrine did not refine risk prediction over a model including the Seattle Heart Failure Model and BNP [59]. In addition to catecholamines, secretory granules of neuroendocrine cells contain chromogranin A and B, whose production increases proportionally to HF severity [60, 61]. In a small study, chromogranin A emerged as a good predictor of mortality in patients with chronic HF [60].

## PRA

In a study of 996 patients with chronic HF, plasma renin activity (PRA) predicted cardiac death independently from NT-proBNP and LVEF [62]. In the Aliskiren Trial on Acute Heart Failure Outcomes (ASTRONAUT) study, low baseline PRA predicted mortality and HF hospitalisation. In this study, PRA reduction during therapy with aliskiren, a direct renin inhibitor, did not predict a better outcome [63].

## ADM

Adrenomedullin (ADM) is a hormone synthesized by almost all tissues, but mainly by the adrenal medulla, heart, lungs, and kidneys, in response to volume or pressure overload. ADM has vasodilatory, natriuretic, inotropic, and cardioprotective effects [64]. Plasma ADM increases in HF, but its dosage is complicated by its short half-life and binding to carrier proteins. A fragment of its precursor, the mid-regional pro-ADM (MR-proADM), is more easily dosed and has been tested as a prognostic marker of HF [64]. In the BACH study, MR-proADM was a better predictor of 90-day survival than BNP in acute HF (accuracy 74% vs. 62%, *p* < 0.001) [42]. This was confirmed in a sub-analysis of the PRIDE study, where MR-proADM was the best predictor of 1-year death, while MR-proANP and NT-proBNP showed a better prognostic performance after 1 year from the diagnosis of acute HF [43]. In 297 patients with chronic HF included in the Australia-New Zealand Heart Failure Study, MR-proADM above the median predicted an increased risk of death (relative risk [RR] 3.92 [1.76–8.7]) and HF hospitalization (RR 2.4 [1.3–4.5]) at 1.5 years, regardless of clinical and echocardiographic parameters [65]. In another study on 501 patients with chronic HF, MR-proADM was a good predictor of 1-year survival with a similar AUC than NT-proBNP (*p* = 0.3) [66]. Although these data support the use of MR-proADM as a prognostic marker, at least for short-term risk stratification, there are some limitations that prevent its use in the clinical practice. Indeed, MR-proADM is not a cardiac specific biomarker, since it is expressed at multiple sites in the body, and factors that may influence its interpretation are yet to be fully characterized.

### Copeptin

Vasopressin is a hormone with antidiuretic and vasoconstrictive activities released by the hypothalamus in response to hyperosmolarity or hypovolemia [67]. In HF, vasopressin is disproportionately increased because of increased baroreceptor stimulation due to a reduced cardiac output simulating a hypovolemic state [67]. The C-terminal fragment of pro-vasopressin is named copeptin, and is more easily dosed than vasopressin [68]. In the BACH study, patients with elevated copeptin had greater lung and peripheral congestion and higher 3-month mortality (HR 3.85 [1.83–8.09]; *p* < 0.001), especially those with hyponatraemia [68]. In a meta-analysis of 4473 patients with acute and chronic HF, copeptin was a good predictor of death from all causes (RR 2.64 [2.09–3.32]), with a similar performance than NT-proBNP [69].

## ET-1

Endothelin-1 (ET-1) is produced by the vascular endothelium in response to shear stress, neuronal stimulation, and inflammation. ET-1 exerts vasoconstrictive, proinflammatory, prooxidative actions and promotes cardiac remodelling. The endothelium releases a precursor, big ET-1, whose C-terminal fragment is then cleaved [70]. In a sub-analysis of the Acute Study of Clinical Effectiveness of Nesiritide in Decompensated Heart Failure (ASCEND-HF), baseline ET-1 correlated with adverse events during hospitalization and 3-month death in patients hospitalized for acute HF, with an additive prognostic value over NT-proBNP [71]. In 115 patients with chronic HF, ET-1 improved the prognostic performance of a model including clinical variables and other biomarkers such as NT-proBNP, hs-TnI, and sST2 [72]. The percentage of time spent with ET-1 ≤ 5.9 pg/mL (corresponding to the 75th percentile) predicted a lower rate of cardiovascular events (odds ratio [OR] 0.75 [0.62–0.91]). Similarly, patients with a reduction in ET-1 over time had a better outcome [72].

## Urocortin-1

Urocortin-1 is a member of the corticotropin-releasing factor family. It is mainly produced in the central nervous system, but also in the heart and other tissues including. Urocortin-1 has vasodilating, inotropic, and cardioprotective effects [73]. Plasma urocortin-1 increases in HF, but this biomarker does not appear to have any additional diagnostic or prognostic value to NT-proBNP [73].

## Biomarkers of cardiac damage

### Cardiac troponins

Elevated cardiac troponins (TnT and TnI) are observed in the majority of patients with acute or chronic HF, especially when measured through high-sensitivity assays. Elevated troponin levels mainly reflect the progressive death of cardiomyocytes, but chronic release of cytoplasmic vesicles (blebs) containing cellular material has also been demonstrated. Possible mechanisms are an imbalance between oxygen demand and supply, particularly in the subendocardial layers, and direct myocardial damage due to neurohormonal activation, inflammation and oxidative stress [74].

Troponins are very effective in prognostic stratification of acute HF. In a study of 144 patients with acute HF, more than 99% of patients had hs-TnI levels above the 99th percentile of the reference population. Furthermore, an hs-TnI > 23 ng/L was associated with an increased risk of hospitalisation or death, which was significantly higher in those with an increase in hs-TnI during hospitalization, compared to those in which hs-TnI remained stable or decreased over time [75]. In 202 patients with acute HF, Pascual-Figal et al. found hs-TnT above the 99th percentile in 81% of cases; hs-TnT > 20 ng/L identified patients with an increased risk of death [76]. Admission hs-TnT was predictive of intra-hospital mortality and at 6, 12, and 24 months in 1499 subjects hospitalized for acute HF, albeit with a lower performance than NT-proBNP [77].

In 4053 subjects with chronic HF from the Val-HeFT, hs-TnT was measurable in 10% of cases with traditional dosages and 92% with high-sensitivity assays; hs-TnT was also predictive of all-cause death [78]. In an individual patient data meta-analysis on 9289 subjects with chronic HF, hs-TnT was predictive of all-cause death (HR 1.48 [1.41–1.55]), cardiovascular death (HR 1.40 [1.33–1.48]) and hospitalization for cardiovascular causes (HR 1.42 [1.36–1.49]) even when adjusting for age, gender, HF etiology, LVEF, GFR, and NT-proBNP [79].

ACC/AHA guidelines recommend the dosage of hs-TnT/I on admission in patients hospitalized for HF or those with chronic HF (class I, LOE A) for the purpose of risk stratification [17]. Conversely, ESC guidelines recommend its use only on admission in patients with suspicion of acute HF within a laboratory analysis panel including full blood cell count, electrolytes, renal function, blood glucose, thyroid and liver function (class I, LOE C), with the main goal of excluding an acute coronary syndrome [2] (Table 2).

High-sensitivity assays allow to detect cardiac troponins in 50–80% of subjects from the general population, sometimes with values as high as > 99th percentile, particularly in subjects at risk of developing HF such as the elderly, patients with cardiovascular risk factors or established cardiac disease [74]. In a study on 2794 asymptomatic subjects with > 65 years of age, hs-TnT > 13 ng/L were predictive of HF development (6.4% [5.8–7.2] per 100 person-year; HR 2.48 [2.04–3.00]) and cardiovascular death (4.8% [4.3–5.4] per 100 person-year; HR 2.91 [2.37–3.58]); moreover, an increase in hs-TnT > 50% predicted a worse prognosis (32). Similarly, a meta-analysis of 67,063 asymptomatic subjects showed that those with higher hs-TnI/T (highest vs. lowest tertile) have a greater risk of developing HF (hs-TnT: HR 2.11 [1.69–2.63]; hs-TnI: HR 2.09 [1.53–2.85]) [80].

## Myocardial remodelling, inflammation, and oxidative stress

### sST2

Suppression of tumorigenesis-2 ligand (ST2L) is a member of the Toll-like receptor family binding interleukin 33 (IL-33) [81]. The IL-33/ST2L axis is mainly a signalling mechanism of the immune system, but has also anti-apoptotic, anti-fibrotic and anti-hypertrophic effects in the heart. sST2 acts as a decoy receptor for IL-33, thus blocking these positive effects. sST2 is mainly produced outside of the heart in response to haemodynamic overload, inflammation, and profibrotic stimuli, which are common in HF [81].

Being a non-cardio-specific marker, sST2 cannot be used to diagnose HF, but it is helpful for risk stratification. A meta-analysis of 4835 patients with acute HF found that both admission and discharge sST2 were predictive of all-cause death (HR 2.46 [1.80–3.37] and 2.06 [1.37–3.11], respectively) and cardiovascular death (HR 2.29 [1.41–3.73] and 2.20 [1.48–3.25], respectively), and that discharge sST2 predicted rehospitalization for HF (HR 1.54 [1.03–2.32]) [82]. Repeating sST2 measurements during HF hospitalization is important, as demonstrated in a study on 150 patients with acute HF, where the percent change in sST2 during hospitalization was predictive of 3-month death regardless of BNP or NT-proBNP [83]. Similar results were found by the Translational Initiative on Unique and novel strategies for Management of Patients with Heart failure (TRIUMPH) cohort study on 496 patients with acute HF with 7 measurements during 1 year of follow-up*.* Baseline sST2 was predictive of all-cause death or HF hospitalization (HR for each standard deviation increase of log_2_(ST2): 1.30 [1.08–1.56]), and changes in sST2 across repeated measurements were even more predictive (HR for each standard deviation increase of the log value_2_(ST2): 1.85 [1.02–3.33]), independent from serial NT-proBNP [84].

sST2 has an important role in risk stratification of chronic HF patients, as demonstrated by numerous studies and confirmed in a meta-analysis by our group [85]. The prognostic value of sST2 in chronic HF is independent from NT-proBNP and hs-TnT [85] and is less influenced by age than the other two biomarkers [86]. sST2 has a similar prognostic performance in HFrEF and HFpEF, and is superior to galectin-3 [81]. Additionally, sST2 independently predicts reverse remodelling and has been included in the ST2-R2 score, which includes sST2 < 48 ng/mL (3 points), non-ischemic etiology (5 points), absence of left branch block (4 points), HF duration < 1 year (2 points), LVEF < 24% (1 point) and treatment with beta-blockers (2 points) [81].

ACC/AHA guidelines recommend sST2 measurement for prognostic evaluation of patients with chronic HF [17] (Table 2), while ESC guidelines state that there is not enough evidence to recommend its use in clinical practice [2].

There is still no unanimous consensus on what is the best prognostic cutoff of sST2 in chronic HF. The most commonly used cutoff is 35 ng/mL, while we proposed the value of 28 ng/mL based on an individual patient data meta-analysis [87]. Higher cutoff values have been proposed for risk stratification in acute HF [87].

### Galectin-3

Galectin-3 is a lectin produced by macrophages involved in tissue and fibrous remodelling [88]. Although overexpressed in HF, galectin-3 is not useful to diagnose acute HF. Instead, galectin-3 is a good predictor of re-hospitalization for HF within 1 to 4 months, as demonstrated by a meta-analysis on 902 patients with acute HF [89]. In the GALectin-3 in Acute heart failure (GALA) study, galectin-3 measured on admission was a good predictor of 30-day mortality, but not of 1-year mortality [90]. However, sub-analyses of recent trials such as RELAXin in Acute Heart Failure (RELAX-AHF) and ProBNP Outpatient Tailored Chronic Heart Failure (PROTECT) did not find a predictive value for 6-month death [91].

The prognostic performance of galectin-3 in HFrEF and HFpEF is lower than other molecules such as NT-proBNP or sST2, and largely influenced by renal function [91]. Nonetheless, ACC/AHA guidelines recommend the dosage of galectin-3 for prognostic stratification of patients with chronic HF, with the same LOE than sST2 [17] (Table 2). ESC guidelines do not recommend its use in clinical practice due to the absence of strong evidence [2].

### GDF-15

Growth differentiation factor-15 (GDF-15) is expressed by numerous cell types, including cardiomyocytes, smooth and endothelial muscle cells, when exposed to several stressors. GDF-15 exerts anti-inflammatory and anti-apoptotic effects. Although it is not a cardiac-specific marker, the increase in plasma GDF-15 seems to have a prognostic role in HF [74]. In a sub-analysis of RELAX-AHF, GDF-15 elevation during a HF hospitalization, but not the admission value, was predictive of cardiovascular death at 180 days or a composite of cardiovascular death, HF hospitalization or kidney failure at 60 days [92]. In a study of 455 patients with chronic HF (median follow-up of 40 months), GDF-15 predicted mortality independent of clinical and laboratory variables including NT-proBNP (HR for an increase of 1 unit in the natural logarithm scale: 2.26 [1.52–3.37]) [93]. Accordingly, a post hoc analysis of Val-HeFT showed that the variation of GDF-15 over 1 year remained an independent predictor of death (HR for changes in GDF-15 at 12 months as a continuous variable: 1.01 [1.00–1.02]), even when accounting for clinical variables, BNP, CRP, hs-TnT, and their changes over time [94]. Unlike NT-proBNP, GDF-15 is not affected by atrial fibrillation, so this biomarker could be particularly useful in these patients [95].

### ECM

The normal turnover of the extracellular matrix (ECM) depends on a balance between the activities of matrix metalloproteinases (MMPs) and their tissue inhibitors (TIMPs). This balance is altered in HF. MMP, TIMP, and many products of ECM degradation have been evaluated as diagnostic or prognostic biomarkers in HF [74]. The combination of increase in MMP-2, TIMP-4, procollagen type III-N-terminal peptide (PIIINP), and decrease in MMP-8 predicted the presence of HFpEF (AUC 0.79) [96]. Circulating levels of these molecules likely reflect the extent of myocardial remodelling, which in turn might be predictive of outcome. In a sub-analysis of the Randomized Aldactone Evaluation Study (RALES), PIIINP > 3.85 μg/L was predictive of adverse outcomes, and a reduction in PIIINP was observed only in patients on spironolactone, confirming the antifibrotic effect of this drug. In addition, the prognostic benefit of spironolactone therapy was significant only in subjects with high basal levels of collagen degradation products [97]. Similarly, in a sub-analysis of the Prospective Comparison of ARNI With ARB Global Outcomes in HF With Preserved Ejection Fraction (PARAGON-HF), treatment with sacubitril/valsartan led to a significant reduction of some degradation products such as TIMP-1 and PIIINP at a 16-month follow-up, compared to valsartan [98].

#### Biomarkers of inflammation and oxidative stress

Inflammation in HF may be triggered by direct cardiomyocyte damage (e.g., because of ischemia or pressure overload), or may reflect a systemic inflammatory state related to comorbidities. The latter mechanism seems crucial in HFpEF [99]. The first demonstration of a CRP elevation in HF dates back to 1956 [8]. Since then, numerous studies have investigated this association, highlighting the prognostic value of CRP, tumor necrosis factor alpha (TNFα), and interleukin-6 (IL-6) in chronic HF [99]. The elevation of these 3 biomarkers has also been associated with an increased risk of HF development in elderly subjects [100, 101]. Recently, cancer antigen 125 (CA-125) has attracted some attention. CA-125 is a glycoprotein synthesized by mesothelial cells in response to increased hydrostatic pressure and/or inflammation. It is widely used as a biomarker for screening and prognostic stratification of many tumors, in particular ovarian cancer [102]. CA-125 levels correlate with signs and symptoms of congestion in HF, and a recent multicentre study in patients with worsening HF showed an association with mortality and risk of HF hospitalization at 1 year [102].

Mitochondrial dysfunction is typical of HF and leads to an increased production of reactive oxygen species (ROS) and a damage to cellular structures [99]. The inherent instability of ROS makes their measurement difficult. However, molecules that interact with ROS, including antioxidants, are good indicators of the oxidative status. The most interesting results in terms of risk stratification in HF have been obtained with myeloperoxidase (MPO) and, to a lesser extent, uric acid [99]. MPO is released by leukocytes as part of the inflammatory response. Plasma MPO increases in chronic HF and is associated with NYHA class and BNP [103]. In another study on 667 patients with acute dyspnea, MPO was equally high in subjects with non-cardiac and cardiac dyspnea, but predicted 1-year mortality regardless of BNP [104].

## Comorbidities

### Kidney dysfunction

Creatinine, azotaemia, and GFR are commonly used in clinical practice to monitor the effects of HF therapies, particularly diuretics. The same parameters are also useful prognostic markers. For example, a study by our group on 9289 patients with chronic HF showed that GFR had an additive prognostic value over NT-proBNP and hs-TnT [105]. It has been suggested that cystatin C, a protease cysteine inhibitor with ubiquitous expression whose clearance is entirely dependent on glomerular filtration, may be an outcome predictor in acute [106] and chronic [107] HF, independent from NPs and potentially superior to creatinine [107].

Cardiovascular and renal systems are functionally interconnected, which explains why indicators of renal damage have been evaluated as HF biomarkers. The most promising molecule is neutrophil gelatinase-associated lipocalin (NGAL), an iron-binding protein released by neutrophils and epithelial cells in response to acute renal damage and inflammation. In a study on 121 patients with acute HF, NGAL levels > 167.5 ng/mL (75th percentile) were associated with a 2.7-fold higher risk of death and a 2.9-fold higher risk of death or hospitalization [108]. In chronic HF, kidney injury molecule*-*1 (KIM-1) and N-acetyl-β-(D)-glucosaminidase (NAG), two molecules expressed by proximal renal tubular cells, were associated with NYHA class and inversely correlated with LVEF. These molecules have been proposed as additional markers of cardio-renal syndrome and outcome in HF [109]. In a cohort of 2,130 patients of the *Gruppo Italiano per lo Studio della Sopravvivenza nell’Insufficienza Cardiaca* (GISSI-HF) study, NGAL, KIM-1, and NAG were independently associated with the combined endpoint of death or HF hospitalization, even in patients with normal renal function [110].

Fibroblast growth factor 23 (FGF-23) is expressed mainly by osteocytes and osteoblasts, and its main targets are the kidneys and parathyroids. FGF-23 was initially implicated in the progression of CKD, and seems to play a role in the development of cardiac hypertrophy and HF [111]. FGF-23 is expressed by cardiomyocytes and enhances RAAS activation through various mechanisms. FGF-23 could then provide a connection between kidney damage, altered bone mineral metabolism and cardiovascular remodelling [111]. FGF-23 levels are related to NYHA class and circulating NPs, and might hold prognostic significance in HFrEF or HFpEF [112].

### Liver dysfunction

Liver dysfunction is common in advanced HF, mostly because of venous congestion due to right ventricular dysfunction. Elevation of transaminases and bilirubin and hypoalbuminemia are able to stratify the risk of patients with acute or chronic HF [113, 114].

### Iron deficiency

Besides worsening HF symptoms, anemia is an established predictor of adverse outcome in patients with acute [115] or chronic HF [116]. Iron deficiency (defined as ferritin < 100 μg/L or 100–300 μg/L with transferrin saturation < 20%) is very common in chronic HF (40–70%), even in the absence of anemia, and predicts a worse prognosis (HR 1.42 [1.14–1.77]) independent from NYHA class, LVEF, kidney function, and NT-proBNP [117]. Randomized clinical trials have shown that correction of iron deficiency in patients with HFrEF improves exercise ability, symptoms, and quality of life [118].

### Endocrine and metabolic alterations

Thyroid dysfunction can manifest itself as subclinical hypothyroidism or low T3 syndrome, is common in patients with chronic HF, and is associated with more severe symptoms and a worse prognosis [119, 120]. Serum and salivary cortisol levels are often elevated in patients with chronic HF and have been associated with increased mortality [121, 122]. Adipokines are a class of hormones involved in regulating carbohydrate metabolism and fatty acids. Adiponectin, a protein secreted inversely proportional to the percentage of body fat, is the most studied so far in HF. Adiponectin increases in chronic HF and seems to predict a worse outcome [123, 124].

## Multi-marker strategies

Multi-marker strategies reflecting different HF pathways could allow to better understand the disease phenotype of each individual patient, and possibly design a tailored therapy. For example, demonstrating an increase of a specific biomarker might prompt the start or up-titration of a therapy able to counteract the mechanisms leading to such elevation. Nonetheless, this approach has never been tested in randomized controlled trials.

Several studies have evaluated multi-marker scores for risk stratification in acute [125] and chronic HF (59), and for risk prediction of incident HF in the general population [126, 127]. For example, we evaluated the measurement of NT-proBNP and hs-TnT with prognostic thresholds stratified by GFR for risk prediction in chronic HF [105]. Many other multi-marker strategies have been proposed [74, 128, 129]. In most studies, the biomarkers were arbitrarily selected. Conversely, in a recent sub-analysis of the Treatment of Preserved Cardiac Function Heart Failure With an Aldosterone Antagonist (TOPCAT) study, a machine-learning approach was pursued to generate a multi-marker panel (from 49 available analytes) able to predict a composite of all-cause death or HF hospitalization in patients with HFpEF [130].

Few biomarkers have shown an additive prognostic value to NPs. They include hs-TnI/T and sST2, and to a lesser extent galectin-3, GDF-15 and some markers of renal dysfunction [74]. ACC/AHA guidelines suggest that a combination of biomarkers (in particular NPs, sST2, galectin-3, and hs-TnI/T) may be more informative than individual biomarkers for risk stratification [17]. Finally, the integration of different biomarkers with clinical variables and imaging characteristics is an interesting, but largely unexplored, perspective.

## -Omics

Several -omics exist: genomics, study of genes and their function; epigenomics, analysis of changes in the genetic code that influence their expression; transcriptomics, study of all RNA transcribed from a given genome; proteomics, evaluation of all proteins present in a cell or tissue; metabolomics, measurement of cellular metabolism products [131]. The main application of genetic testing in HF has been the identification of monogenic cardiomyopathies. In these cases, the identification of a culprit gene can influence therapeutic choices and lead to screening of family members. However, HF is most commonly the result of an interaction between genetic susceptibility and environmental exposure [131]. Genome-wide association studies have attempted to explore the association between specific single-nucleotide polymorphism (SNP) and the onset/progression of HF, with limited results [132]. Recent studies have also sought to identify epigenetic modifications potentially associated with HF development [133].

Transcriptomic studies have identified specific patterns of gene expression associated with different HF phenotypes (e.g., HFpEF vs. HFrEF) [134]. Many studies focused on the evaluation of microRNAs (miRNAs), short non-coding RNA able to influence gene expression at the post-transcriptional level. Several miRNAs are chemically stable and can be easily dosed in the plasma. Circulating levels of more than 30 miRNAs increase or decrease in patients with HF. miRNA panels seem able to accurately discriminate HFpEF from HFrEF [135]. However, strong evidence on the role of specific miRNAs for diagnosis, risk stratification or guide to treatment is currently lacking.

A proteomic approach showed that quiescin Q6, a protein involved in the formation of disulphide bonds, allows to discriminate acute HF from no-cardiac dyspnea [136]. Proteomic profiling has also been used to identify predictors of incident HF. For example, in a study including two community-based prospective cohorts of elderly individuals without HF, a proteomic analysis found that 9 proteins involved in apoptosis, inflammation, matrix remodelling, and fibrinolysis improved prediction of incident HF over a model including established risk factors (age, ethnicity, gender, heart rate, systolic blood pressure, anti-hypertensive medications, diabetes, coronary heart disease, smoking status, body mass index), but not when NT-proBNP was added to the model [137]. Moreover, Adamo et al. recently employed a proteomic approach to define proteomic signatures unique to specific HF phenotypes, such as HFrEF, HFpEF, and HFmrEF with improved or unchanged LVEF, ischemic and non-ischemic HF [138].

Some authors have employed metabolomic profiling to identify novel biomarkers or multi-marker panels associated with incident HF or HF prognosis [131]. Using a metabolomic approach, Hunter et al. found that plasma long-chain acylcarnitine concentration was significantly higher in HF patients compared to controls, and even discriminated HFrEF from HFpEF, being higher in HFrEF [139]. Another metabolomic study demonstrated that circulating long-chain acylcarnitine was independently associated with the risk for hospital readmission and mortality in chronic HF; in addition, long-chain acylcarnitine concentration decreased after LV assist device placement in a subgroup of patients with end-stage HF [140]. As for multi-marker approaches, in a study on 1,032 HFrEF patients, a 13-metabolite profile was defined and then validated as a predictor of mortality, improving survival models including established clinical risk factors and even NPs [141].

## Conclusions

BNP and NT-proBNP are the gold standard in prognostic diagnosis and stratification of HF and their use is recommended by both ESC and ACC/AHA guidelines. NP measurement may allow to identify patients with subclinical LV dysfunction, thus allowing preventive measures to slow down the progression to clinical HF. The role of NPs as guides to treatment remains unclear. BNP and NT-proBNP have different diagnostic cutoffs, and NP concentrations should be interpreted on the light of many factors such as age, kidney function, arrhythmia, and obesity, among others. Other biomarkers can provide additional information to NPs. Many biomarkers have been evaluated, linked to specific mechanisms of neurohormonal activation, myocardial damage, cardiac remodelling, inflammation/oxidative stress, and comorbidities. High-sensitivity troponins and sST2 are currently the most promising biomarkers as additive tools to BNP and NT-proBNP for prognostic stratification of HF. Multi-marker panels or scores and the different -omic techniques represent other intriguing perspectives for future research.
